# Artificial Intelligence Models for Predicting Outcomes in Spinal Metastasis: A Systematic Review and Meta-Analysis

**DOI:** 10.3390/jcm14165885

**Published:** 2025-08-20

**Authors:** Vivek Sanker, Prachi Dawer, Alexander Thaller, Zhikai Li, Philip Heesen, Srinath Hariharan, Emil O. R. Nordin, Maria Jose Cavagnaro, John Ratliff, Atman Desai

**Affiliations:** 1 Department of Neurosurgery, Stanford University, Palo Alto, CA 94305, USA; hsrinath@stanford.edu (S.H.); enordin@stanford.edu (E.O.R.N.); mjcava@stanford.edu (M.J.C.); jratliff@stanford.edu (J.R.); atman@stanford.edu (A.D.); 2 Department of Neurosurgery, University College of Medical Sciences, New Delhi 110095, India; prachidawar59@gmail.com; 3 Department of Neurosurgery, Medical University of Graz, 8010 Graz, ST, Austria; alexander.thaller@medunigraz.at; 4 Department of Clinical Neurosciences, Addenbrooke’s Hospital, Cambridge CB2 0QQ, UK; zl498@cam.ac.uk; 5 Faculty of Medicine, University of Zurich, 8006 Zurich, Switzerland; heesenphilip99@gmail.com

**Keywords:** artificial intelligence, machine learning, deep learning, spine metastasis, complications

## Abstract

**Background:** Spinal metastases can cause significant impairment of neurological function and quality of life. Hence, personalized clinical decision-making based on prognosis and likely outcome is desirable. The effectiveness of AI in predicting complications and treatment outcomes for patients with spinal metastases is assessed. **Methods:** A thorough search was carried out through the PubMed, Scopus, Web of Science, Embase, and Cochrane databases up until 27 January 2025. Included were studies that used AI-based models to predict outcomes for adult patients with spinal metastases. Three reviewers independently extracted the data, and screening was conducted in accordance with PRISMA principles. AUC results were pooled using a random-effects model, and the PROBAST program was used to evaluate the study’s quality. **Results:** Included were 47 articles totaling 25,790 patients. For training, internal validation, and external validation, the weighted average AUCs were 0.762, 0.876, and 0.810, respectively. The Skeletal Oncology Research Group machine learning algorithms (SORG-MLAs) were the ones externally validated the most, continuously producing AUCs > 0.84 for 90-day and 1-year mortality. Models based on radiomics showed promise in preoperative planning, especially for outcomes of radiation and concealed blood loss. Most research concentrated on breast, lung, and prostate malignancies, which limited its applicability to less common tumors. **Conclusions:** AI models have shown reasonable accuracy in predicting mortality, ambulatory status, blood loss, and surgical complications in patients with spinal metastases. Wider implementation necessitates additional validation, data standardization, and ethical and regulatory framework evaluation. Future work should concentrate on creating multimodal, hybrid models and assessing their practical applications.

## 1. Introduction

The spine is one of the most common sites of metastasis, after the lung and the liver. In patients with systemic cancer, approximately more than half develop spinal metastases, and approximately 10% are symptomatic [1]. Surgery can significantly improve quality of life in selected patients [2], and overall treatments have advanced in recent years to enhance overall clinical results and survival [3]. However, the likelihood of good outcomes must always be weighed against the risks of complications and the economic costs in each individual case [4].

Artificial intelligence (AI) is emerging as a potentially powerful tool to enhance clinical decision-making through analysis of large datasets to predict individual patient outcomes and risks, through machine learning and deep learning algorithms.

This systematic review’s main goal was to assess the state of artificial intelligence (AI) models created to forecast outcomes and problems for patients who have spinal metastases. The degree to which these models included explainability and interpretability—two crucial components for clinical confidence and the practical application of AI tools—was specifically examined in addition to summarizing performance measures. The following definition of the primary clinical outcomes was made to guarantee uniformity between studies:

- Survival: At the longest or most precise follow-up available, it is reported as either overall survival (OS) or progression-free survival (PFS). PFS is the period of time until disease progression or death, whereas OS is the period of time from diagnosis or the start of therapy to death from any cause.
- Ambulatory status: Usually classified as either ambulatory or non-ambulatory, this refers to the patient’s capacity to walk on their own or with the use of assistive technology.
- Complications: Contains any unfavorable events that occur during or after surgery, such as bleeding, infection, thrombosis, or neurological decline. Standard classification systems (e.g., Clavien–Dindo) were used to stratify these by severity whenever possible.

## 2. Materials and Methods

### 2.1. Ethical Review

Ethical review and approval were waived for this study due to it being a systematic review of previously published data that did not involve human participants or the collection of new data.

### 2.2. Search Strategy

We searched PubMed, Scopus, Web of Science Advance, Cochrane, and Embase (Ovid) databases to identify relevant studies, using a search query with specific keywords like ‘spine metastases’, ‘artificial intelligence’, ‘machine learning’, ‘deep learning’, and ‘outcomes’ (Supplementary Table S1). The population under consideration included adults with spinal metastases. The objective was to identify studies reporting the use of AI/deep learning (DL) models in predicting treatment and outcome prediction in spinal metastases [5].

Irrelevant articles, such as studies unrelated to spinal metastases and those purely investigating primary spinal tumors, were excluded. Animal studies, reviews, and non-original research articles were also excluded from our analysis to ensure the inclusion of primary research data relevant to our objective. The electronic search ranged from the period’s earliest available date up to 27 January 2025 [5].

### 2.3. Screening of Studies

**Figure 1:**
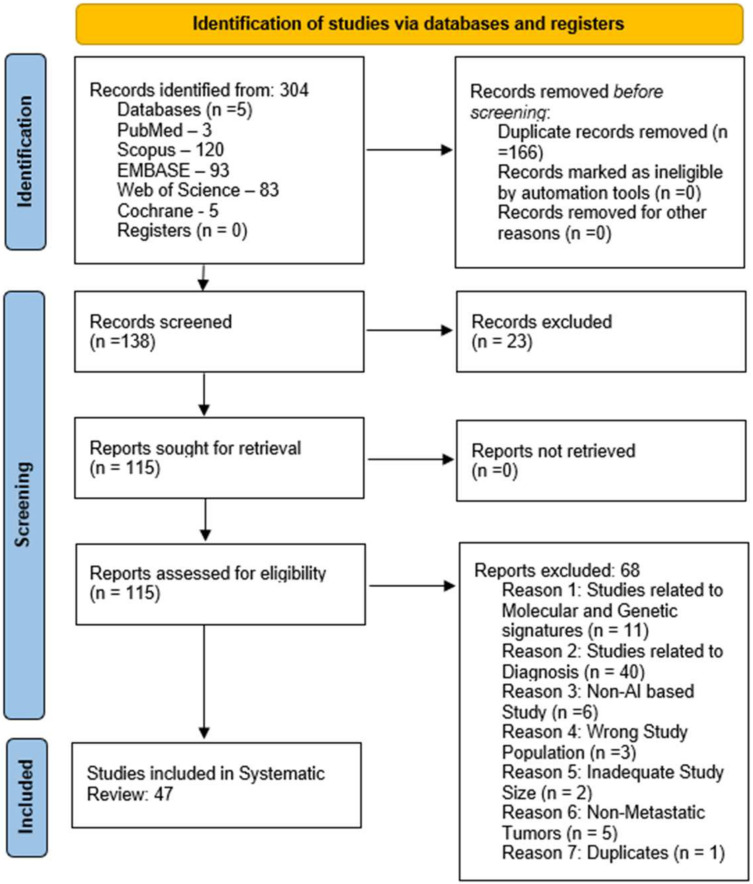
A PRISMA flow diagram is presented to illustrate the screening of studies.

Each study’s title and abstract were screened for relevance before proceeding to full-text screening, which was independently assessed by two reviewers (PD and VS). Any discrepancies were addressed through consultation with a third reviewer (SH). This review adhered to the PRISMA (Preferred Reporting Items for Systematic Reviews and Meta-Analyses) guidelines but was not registered on the PROSPERO international prospective register of systematic reviews (Figure 1).

### 2.4. Data Extraction

Three independent authors (PD, VS, and AT) extracted relevant data from the included studies. The data collected included study design, participant demographics, and the number of participants with respective outcomes and complications. Discrepancies in data extraction were resolved through consensus [5].

### 2.5. Data Analysis

Relevant variables were extracted from each of the included articles, such as the primary tumor type, cohort size, and prediction model performance matrices: area under the receiver operating characteristic curve (AUC) and type of validation (internal or external validation). The weighted average of the AUC was calculated. All statistical analyses were conducted using Excel, R Statistical Software, version 4.3.1, and Python, version 3.13.3.

We conducted a random-effects meta-analysis using restricted maximum likelihood (REML) estimation to account for both within-study and between-study variability. This approach assumes that the true effect size may vary across studies due to underlying differences in study populations, methodologies, or settings. REML was used to estimate the between-study variance (τ^2^), providing an unbiased and efficient estimate of heterogeneity. Pooled effect estimates were calculated as weighted averages of the individual study effects, with weights derived from both the within-study variance and the estimated between-study variance. The Standardized Mean Difference (SMD) along with the 95% confidence interval (CI) was used to compare continuous performance metrics like AUC between studies after having a pooled estimate.

### 2.6. Quality Assessment

The quality assessment was performed using the PROBAST (Prediction model Risk of Bias Assessment Tool (Supplementary Figures S1 and S2).

PROBAST is designed for assessing the risk of bias and applicability in studies that develop, validate, or update predictive models. It is a structured tool that assesses four domains:

Participants: evaluating whether the data sources or patient samples used for training and testing are appropriate and representative of the clinical population. Predictors: ensuring that input data or predictors are well defined and appropriately measured. Outcome: ensuring that the outcomes (e.g., model predictions, decisions) are clearly defined and relevant to clinical scenarios. Analysis: evaluating whether the model performance metrics, training/validation processes, and statistical analysis methods are robust and unbiased.

In the Participants Section, 32 studies were flagged as having low risk of bias (68%), whilst 15 studies were flagged as having unclear risk/some concerns (32%). In the Predictors Section, 23 studies were flagged as having low risk of bias (49%), 16 studies flagged as having unclear risk/some concerns (34%), and 8 studies flagged as having high risk (17%). In the Outcome Section, 18 studies were flagged as having low risk of bias (38%), 16 as having unclear risk/some concerns (34%), and 13 as having high risk (28%). In the Analysis Section, 3 studies were flagged as having low risk of bias (6%), 15 studies flagged as having unclear risk/some concerns (32%), and 29 flagged as having high risk (62%).

## 3. Results

This review encompasses 47 studies published between 2016 and 2025, including a total of 26,038 patients with a median of 269 patients per study, ranging from 30 to 2786 patients (Table 1).

**Figure 2:**
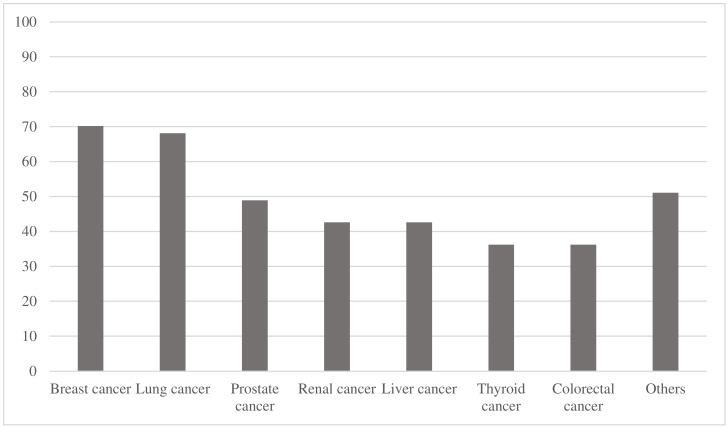
The percentage of how many studies feature specific primary tumor types is presented.

Among the 47 studies, the three most common primary tumor types were breast cancer, lung cancer and prostate cancer, reported in 33 (70.2%), 32 (68.1%), and 23 (48.9%) studies, respectively (Table 2 and Figure 2). In contrast, neuroendocrine tumors, bladder cancer and esophageal cancer were the least common, each reported in three (6.4%), three (6.4%), and four (8.5%) studies, respectively (Table 2).

Five of the forty-seven studies reported AUC values for the established models during training of the model (Table 3), eighteen for internal validation (Table 4), and fourteen for external validation (Table 5).

The weighted average AUC value among the five studies that reported AUC values and corresponding 95% confidence intervals for the training of the established models is 0.762 (95% CI: 0.704–0.717). Wherever 95% confidence intervals were not reported, the weighted average of the reported 95% confidence intervals was used.

The weighted average AUC value among the 18 studies that reported AUC values and corresponding 95% confidence intervals for internal validation of the established models is 0.876 (95% CI: 0.871–0.881). Wherever 95% confidence intervals were not reported, the weighted average of the reported 95% confidence intervals was used.

The weighted average AUC value among the eight studies that reported AUC values and corresponding 95% confidence intervals for external validation of the established models is 0.810 (95% CI: 0.803–0.816).

### Meta-Analysis of the SORG-MLA Model

**Figure 3:**
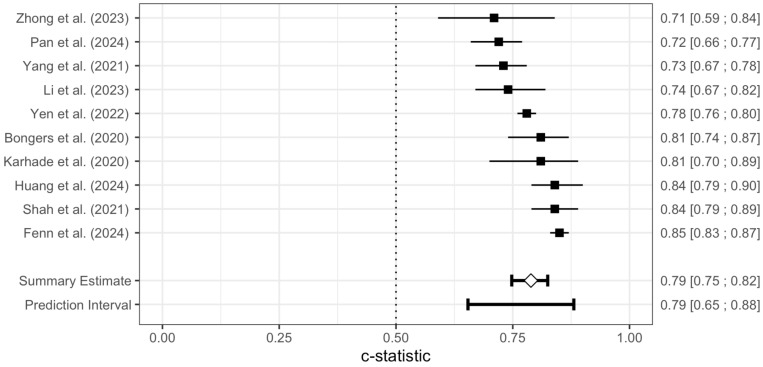
A Forest plot of the meta-analysis of the AUC for the SORG-MLA model for 90-day survival is shown. The AUC and 95% Confidence Intervals of each independent model validation are depicted. The diamond represents the pooled AUC along with a 95% Confidence Interval. A prediction interval is presented [26,27,28,29,31,32,33,34,35,36].

**Figure 4:**
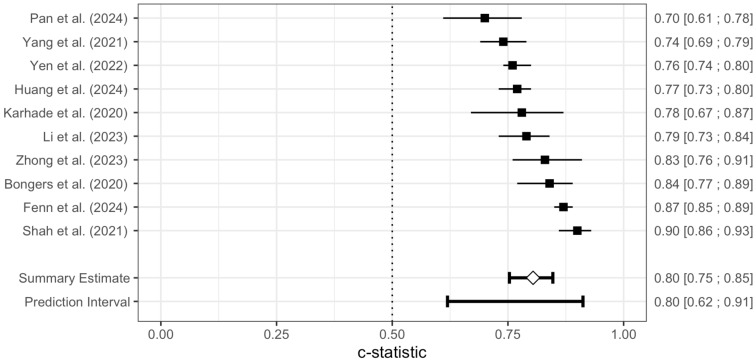
Forest plot of the meta-analysis of the AUC for the SORG-MLA model for 1-year survival. The AUC and 95% Confidence Intervals of each independent model validation are depicted. The diamond represents the pooled AUC along with a 95% Confidence Interval. A prediction interval is presented [26,27,28,29,31,32,33,34,35,36].

The pooled AUC of the SORG-MLA model for 90-day survival was 0.79 (95% CI: 0.75–0.82) and for 1-year survival 0.80 (95% CI: 0.75–0.85). The prediction intervals ranged from 0.65 to 0.88 and from 0.62 to 0.91, respectively. Figure 3 and Figure 4 are forest plots of the 90-day survival and 1-year survival.

## 4. Discussion

The integration of artificial intelligence (AI) into clinical oncology is revolutionizing the care of patients with spinal metastases. Here, data were combined from 47 studies with a pool of >25,000 patients with a range of models presented for a breadth of clinical applications, from predicting surgical mortality and complications to estimating occult blood loss and assessing perioperative functional status. The heterogeneity of use-cases highlights the versatility of AI for addressing different facets of managing spinal metastases. Notably, prediction models demonstrated strong discriminative performance with pooled AUC values of 0.762, 0.876, and 0.810 for training, internal, and external validation, respectively, supporting the reasonable accuracy and generalizability of these models across multiple datasets (Table 3, Table 4 and Table 5). Additionally, the predominance of certain primary tumor types, including breast, lung, and prostate, within the included studies reflects their real-world contribution to spinal metastases. While this focus ensures relevance to a large patient population, it also raises questions about generalizability of these models to rarer malignancies which might be underrepresented, including neuroendocrine or esophageal sources. There is a need for more balanced datasets and the inclusion of between- and within-subgroup analyses to account for tumor-type-specific variability in outcomes and treatment response.

Reproducibility remains a fundamental challenge in radiomics-based models due to variability in image acquisition protocols, reconstruction parameters, and scanner types across institutions. These inconsistencies can substantially alter extracted radiomic features, thereby affecting model performance when applied outside the development cohort [37]. Our review noted that only a minority of studies implemented harmonization techniques such as image resampling, intensity normalization, or ComBat-based feature adjustment, which aim to mitigate site-specific variability. The lack of standardization across studies limits generalizability and may contribute to overfitting or unstable performance in external validation cohorts [37]. Future research should prioritize standardized radiomics pipelines and transparent reporting of acquisition parameters to enable reliable replication and multi-center deployment of radiomics-based predictive tools.

Crucially, survival prediction emerged as the most extensively studied application of AI in spinal metastasis, with several model algorithms, especially those developed by the Skeletal Oncology Research Group (SORG), subject to both internal and international external validation. The consistent performance of these models across different populations and healthcare infrastructures supports their utility as decision-support tools [23,24,30,33]. For instance, models predicting 90-day and 1-year mortality yielded AUCs approaching or exceeding 0.84 in many studies, making a strong case for these tools to be used in preoperative planning or when evaluating eligibility for aggressive intervention [21,23,24,35,36]. Despite this, all studies that externally evaluated the SORG-MLA were retrospective in their design, thereby limiting the real-world significance of the pooled AUCs. Therefore, the realization of real-world impact studies is encouraged to assess clinical endpoints such as changes in treatment decisions and improved survival.

Although AI models’ prognostic abilities in spinal metastases have shown promise, the discipline is still struggling with the crucial problems of interpretability and explainability. The safe and moral incorporation of AI into healthcare decision-making depends on these factors. The fact that many of the evaluated research rely on opaque, sophisticated algorithms, including deep neural networks and ensemble-based models like XGBoost, random forest, CatBoost, or Gaussian processes, without providing clear reasoning routes, is a major drawback [12,13,15,17,18,20,21]. A model must be interpretable in order to promote patient acceptance, clinician trust, and regulatory compliance in the therapeutic setting, where decisions have significant consequences [3,10].

Because it is difficult to understand how input features affect predictions, there is rising worry that “black box” models might be dangerous for patient care [10,15,17]. However, only a small percentage of the papers we examined used explainable AI (XAI) methods to help physicians and stakeholders understand the model’s logic, such as SHAP (Shapley Additive Explanations), LIME (Local Interpretable Model-Agnostic Explanations), or decision-path visualizations. For example, there was little investigation into the methods by which high-performing models created with random forest [10,22], support vector machines [13], and deep learning [19] arrived at their predictions.

Adoption in multidisciplinary decision-making settings [5], when physicians are held responsible for defending treatment decisions, may be constrained by this lack of openness. It will be crucial to include model interpretability tools (such as attention heatmaps and feature significance plots) into upcoming development workflows. AI in spine cancer cannot be meaningfully implemented without clinician-centered design, explainable model architecture, and cross-validation across a variety of datasets [3,25].

Adoption and clinical implementation of AI models is largely dependent on the ease of use and availability of such models. We identified six studies [12,15,21,23,24,27] that deployed a user-friendly platform, i.e., web-based applications. Of these studies, only two web-based applications [21,24] were working trouble-free at the time of the creation of this manuscript. The two functioning applications represent the SORG-MLA at different time points, which have been integrated into one website [24]. Such availability builds the foundation on which clinical uptake and regular usage is dependent. Therefore, more models should be made readily available by deploying them via user-friendly platforms, such as websites, mobile apps etc.

## 5. Limitations

We have now added a specific Limitations Section to offer a thorough and open evaluation of the limitations of this research. Regarding data preparation, outcome definitions, validation methods, and reporting standards, the included studies exhibit methodological variability. To reduce direct cross-comparability of model performance, for example, some research used split-sample validation or no validation at all, while others used k-fold cross-validation [14,15,21].

Second, there is significant worry about demographic and regional bias across datasets. The bulk of training cohorts come from middle- to high-income nations like China, Taiwan, and the United States [21,30,31,34], which can restrict the applicability of AI tools to underrepresented groups or environments with limited resources. International external validation studies are available [31,33,34], but few of them specifically address equality, cultural adaptability, or the diversity of healthcare systems when using AI. Further external validation should thus be carried out to ensure applicability and global generalizability of existing models. Ethnic or racial biases are addressed in some studies; however, the homogenous nature of many validation cohorts does not allow for adequate distinctions of model performance between different ethnic or racial groups to be made. Future models should therefore be trained on a heterogenous pool of patients to account for cultural, ethnic, racial, and socioeconomic differences.

Third, while some models showed good discrimination metrics (AUCs > 0.85), we were unable to fully evaluate the clinical value of these tools since calibration metrics and decision-curve analysis were seldom published [23,25,32]. It is noteworthy that the majority of models are still in the research realm and have not yet been included into real-time clinical decision processes or electronic health records (EHRs2). The limited number of studies that documented attempts to create web-based apps or clinician-facing tools [15,38] limited its translational preparedness.

Lastly, the examined literature still lacks sufficient attention to ethical and practical concerns, such as patient privacy, data completeness, interpretability, and openness. For instance, in several studies, missing variables (such as albumin or lymphocyte counts) deteriorated model performance [27], but few models included flexible topologies or robust imputation to deal with such uncertainty. Future multicenter, prospective, inclusive studies that assess the practical performance, equity, and stakeholder acceptance of AI tools in spinal cancer are desperately needed, as these data highlight.

## 6. Conclusions

AI has the potential to advance spinal metastasis care in the domains of outcome prediction and risk stratification and thus enhance precision medicine and streamline clinical workflow to improve patient outcomes. If rigorously validated and ethically deployed, AI has the potential not just to predict outcomes but to transform spinal oncology into a more personalized, equitable, and data-driven discipline. As AI continues to evolve within spinal oncology, its success will depend not only on predictive accuracy but also on transparency, interpretability, and ethical deployment. Future models must prioritize explainability to foster clinician trust, support informed consent, and ensure equitable care across diverse patient populations.

## Figures and Tables

**Table 1:** An overview of the studies analyzed is presented.

Year of publishing (range)	2016–2025
Total number of patients	25,790
Median number of patients	268
Number of patients per study (range)	30–2786

**Table 2:** The frequency of primary tumor types is shown. Studies analyzing multiple primary tumor types have been included in all applicable categories.

Primary Tumor Type	Number of Studies (Percentage)
Breast cancer	33/47 (70.2%)
Lung cancer	32/47 (68.1%)
Prostate cancer	23/47 (48.9%)
Esophageal cancer	4/47 (8.5%)
Bladder cancer	3/47 (6.4%)
Neuroendocrine tumors	3/47 (6.4%)

**Table 3:** AUC (training of each study’s best model).

Study	Output/Prediction	Best-Performing Model	AUC	95% CI
Zhao et al. (2024) [6]	Hidden blood loss in spinal metastasis surgery	MRI-Based Radiomics	0.784	-
Bakhsheshian et al. (2022) [7]	Mortality	Machine Learning Model using ECI ^1^ and Frailty	0.788	-
	Medical complications		0.723	-
Massaad et al. (2022) [8]	1-year mortality	Machine Learning Model using Body Composition and NESMS ^2^	0.73	0.67–0.78
Shi et al. (2022) [9]	Response of osteolytic metastases to chemotherapy	Radiomics (T2WI + ADC_all_)	0.908	0.86–0.96
Massaad et al. (2021) [10]	Postoperative complications	Random Forest to develop MSTFI ^3^	0.62	0.56–0.68

The AUC and corresponding 95% confidence interval of studies reporting AUC values for the established radiomics models for the training of the model is presented. 1 = Elixhauser Comorbidity Index, 2 = New England Spinal Metastasis Score, 3 = Metastatic Spinal Tumor Frailty Index.

**Table 4:** AUC (internal validation of each study’s best model).

Study	Output/Prediction	Best-Performing Model		AUC		95% CI	
Santipas et al. (2024) [11]	Complications after cervical spine metastases surgery	Gradient Boosting		0.939 ^1^	0.873 ^2^	-	-
Cui et al. (2024) [12]	Postoperative ambulatory status	Ensemble Machine Learning combining LR ^6^, eXGBM ^7^, SVM ^8^, RF ^9^, NN ^10^ and DT ^11^		0.911 ^1^		0.854–0.968 ^1^	
Santipas et al. (2024) [13]	30-day preoperative VTE ^5^	Gradient Boosted Trees		0.77 ^1^		-	
	90-day preoperative VTE ^5^	Support Vector Machine		0.72 ^1^		-	
	30-day postoperative VTE ^5^	Gradient Boosted Trees		0.71 ^1^		-	
	90-day postoperative VTE ^5^	Support Vector Machine		0.68 ^1^		-	
Santipas et al. (2024) [14]	90-day survival	CatBoost		0.750 ^1^	0.758 ^2^	-	-
	180-day survival	XGBoost		0.726 ^1^	0.744 ^2^	-	-
	365-day survival	XGBoost		0.731 ^1^	0.693 ^2^	-	
Shi et al. (2024) [15]	Massive intraoperative blood loss	XGBoosting machine (XGBM; Machine Learning)		0.857 ^2^		0.827–0.877 ^2^	
Zhao et al. (2024) [6]	Hidden blood loss	MRI-Based Radiomics		0.744 ^2^		0.576–0.914 ^2^	
Chavalparit et al. (2023) [16]	90-day postoperative ambulatory status	Decision Tree		0.941 ^1^		-	
	180-day postoperative ambulatory status	Extreme Gradient Boosting		0.852 ^1^		-	
Chen et al. (2023) [17]	Treatment outcome after stereotactic body RT ^12^	Gaussian Processes		0.828 ^1^		-	
Gao et al. (2023) [18]	Severe psychological distress	Gradient Boosting Machine (Machine Learning)		0.865 ^2^		0.788–0.941 ^2^	
Hallinan et al. (2022) [19]	Grading metastatic epidural spinal cord compression (Bilsky grading (normal/low versus high)	Separated Window Learning (Max fusion model)		0.971 ^2^		0.961–0.981 ^2^	
	Grading metastatic epidural spinal cord compression (Bilsky grading (normal versus low/high)	Separated Window Learning (Spine-window)		0.924 ^2^		0.910–0.938 ^2^	
Jabehdar Maralani et al. (2022) [20]	Response following stereotactic body RT	Decision Tree		0.923 ^1^	0.959 ^2^	-	-
Karhade et al. (2022) [21]	6-week mortality	Elastic-net penalized logistic regression		0.85 ^1^	0.84 ^2^	0.84–0.86 ^1^	0.80–0.88 ^2^
Shi et al. (2022) [9]	Response of osteolytic metastases to chemotherapy	Radiomics (FST2WI + ADC_all_)		0.873 ^2^		0.78–0.96 ^2^	
Gui et al. (2022) [22]	Risk of vertebral compression fracture after stereotactic body RT ^12^	Random Forest		0.878 ^1^		0.832–0.924 ^1^	
Massaad et al. (2021) [10]	Postoperative complications	Random Forest to develop MSTFI ^3^		0.69 ^4^		0.66–0.73 ^4^	
Karhade et al. (2019) [23]	30-day mortality	Bayes Point Machine (Machine Learning)		0.786 ^1^	0.782 ^2^	-	-
Karhade et al. (2019) [24]	90-day mortality	Stochastic gradient boosting		0.83 ^1^	0.83 ^2^	0.81–0.85 ^1^	-
	1-year mortality			0.85 ^1^	0.89 ^2^	0.83–0.87 ^1^	-
Paulino Pereira et al. (2016) [25]	30-day survival	Boosting Algorithm	Nomogram	0.91 ^1^	0.75 ^2^	0.86–0.95 ^1^	0.60–0.89 ^2^
	90-day survival			0.86 ^1^	0.73 ^2^	0.83–0.90 ^1^	0.63–0.83 ^2^
	1-year survival			0.84 ^1^	0.75 ^2^	0.80–0.87 ^1^	0.67–0.84 ^2^

The AUC and corresponding 95% confidence interval of studies reporting AUC values for the established radiomics models for internal validation of the model is presented. 1 = k-fold cross validation, 2 = split sample internal validation, 3 = Metastatic Spinal Tumor Frailty Index, 4 = Internal Bootstrap Validation, 5 = venous thromboembolism, 6 = Logistic Regression, 7 = Extreme Gradient Boosting Machine, 8 = Support Vector Machine, 9 = Random Forest, 10 = Neural Network, 11 = Decision Tree, 12 = Radiotherapy.

**Table 5:** AUC (external validation of each study’s best model).

Study	Output/Prediction	Best Performing Model	AUC	95% CI
Cui et al. (2024) [12]	Postoperative ambulatory status	Ensemble Machine Learning combining LR ^2^, eXGBM ^3^, SVM ^4^, RF ^5^, NN ^6^ and DT ^7^	0.873	0.809–0.936
			0.924	0.890–0.959
Fenn et al. (2024) [26]	90-day mortality	Machine Learning Algorithm (SORG-MLA)	0.85	0.83–0.87
	1-year mortality		0.87	0.85–0.89
Huang et al. (2024) [27]	6-week survival	Machine Learning Algorithm (SORG-MLA)	0.84	0.78–0.89
	90-day survival		0.84	0.79–0.90
	1-year survival		0.77	0.73–0.80
Pan et al. (2024) [28]	42-day survival	Machine Learning Algorithm (SORG-MLA)	0.69	0.63–0.74
	90-day survival		0.72	0.66–0.77
	1-year survival		0.70	0.61–0.78
Shi et al. (2024) [15]	Massive intraoperative blood loss for spinal metastases	XGBoosting machine (XGBM; Machine Learning)	0.809	0.778–0.860
Li et al. (2023) [29]	90-day survival	Machine Learning Algorithm (SORG-MLA)	0.743	0.666–0.817
	180-day survival	Machine Learning Algorithm (Revised Katagiri)	0.761	0.696–0.826
	1-year survival	Machine Learning Algorithm (SORG-MLA)	0.787	0.730–0.838
	2-year survival	Machine Learning Algorithm (Revised Katagiri)	0.779	0.747–0.811
Su et al. (2023) [30]	6-week survival after RT ^1^ only	Machine Learning Algorithm (SORG-MLA)	0.77	0.74–0.79
	6-week survival after surgery		0.84	0.79–0.90
Zhong et al. (2023) [31]	90-day mortality	Machine Learning Algorithm (SORG-MLA)	0.714	0.589–0.839
	1-year mortality		0.832	0.758–0.906
Karhade et al. (2022) [21]	6-week mortality	Elastic-net penalized logistic regression	0.82	0.78–0.85
Yen et al. (2022) [32]	90-day survival	Machine Learning Algorithm (SORG-MLA)	0.78	0.76–0.80
	1-year survival		0.76	0.74–0.78
Shah et al. (2021) [33]	90-day mortality	Machine Learning Algorithm (SORG-MLA)	0.84	0.79–0.89
	1-year mortality		0.90	0.86–0.93
Yang et al. (2021) [34]	90-day mortality	Machine Learning Algorithm (SORG-MLA)	0.73	0.67–0.78
	1-year mortality		0.74	0.69–0.79
Bongers et al. (2020) [35]	90-day mortality	Machine Learning Algorithm (SORG-MLA)	0.81	0.74–0.87
	1-year mortality		0.84	0.77–0.89
Karhade et al. (2020) [36]	90-day mortality	Machine Learning Algorithm (SORG-MLA)	0.81	0.70–0.89
	1-year mortality		0.78	0.67–0.87

The AUC and corresponding 95% confidence interval of studies reporting AUC values for the established radiomics models for external validation of the model is presented. 1 = Radiation Therapy 2 = Logistic Regression, 3 = Extreme Gradient Boosting Machine, 4 = Support Vector Machine, 5 = Random Forest, 6 = Neural Network, 7 = Decision Tree.

## Data Availability

All datasets used for this study are presented in the manuscript and its Supplementary Materials.

## Supplementary Materials

The following supporting information can be downloaded at: https://www.mdpi.com/article/10.3390/jcm14165885/s1, Supplementary Figure S1: Risk of bias. Supplementary Figure S2: Applicability. Supplementary Table S1: Search queries across databases. Supplementary Table S2: Summary of the studies analyzed. References [39,40,41,42,43,44,45,46,47,48,49,50,51,52,53] are cited in the supplementary materials.
