# Supplementary material for: Artificial Intelligence Models for Predicting Outcomes in Spinal Metastasis: A Systematic Review and Meta-Analysis
Source: J Clin Med. 2025 Aug 20;14(16):5885. doi: 10.3390/jcm14165885 (PMC12387370; doi:10.3390/jcm14165885)
Supplement: Supplementary file 1 [file jcm-14-05885-s001.zip › jcm-3721915-supplementary.pdf]

Supplementary Files

Supplementary Figure S1 – Risk of bias

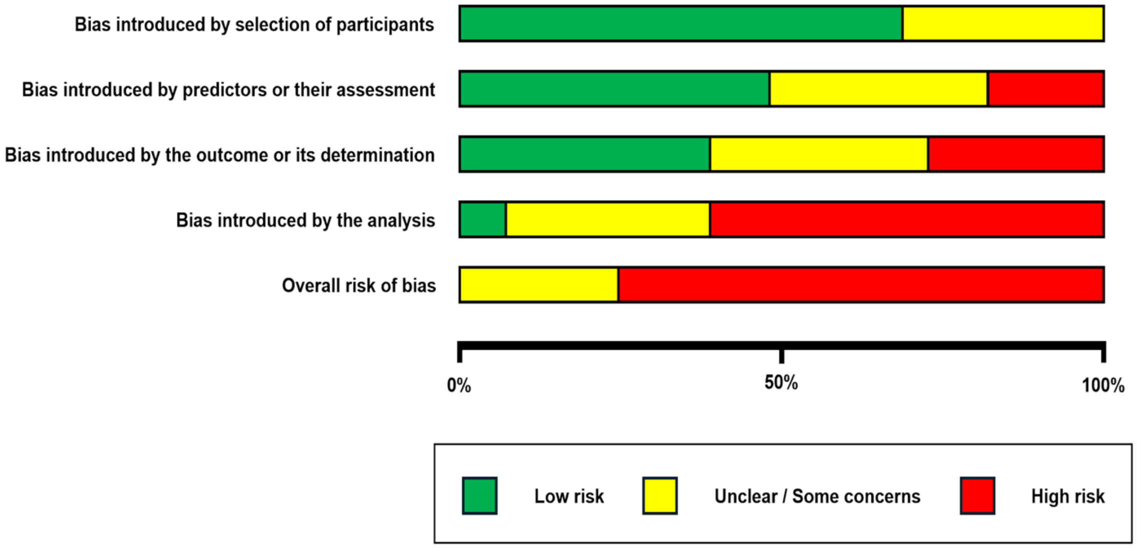

Supplementary Figure S1. A risk of bias analysis is presented.

Supplementary Figure S2 - Applicability

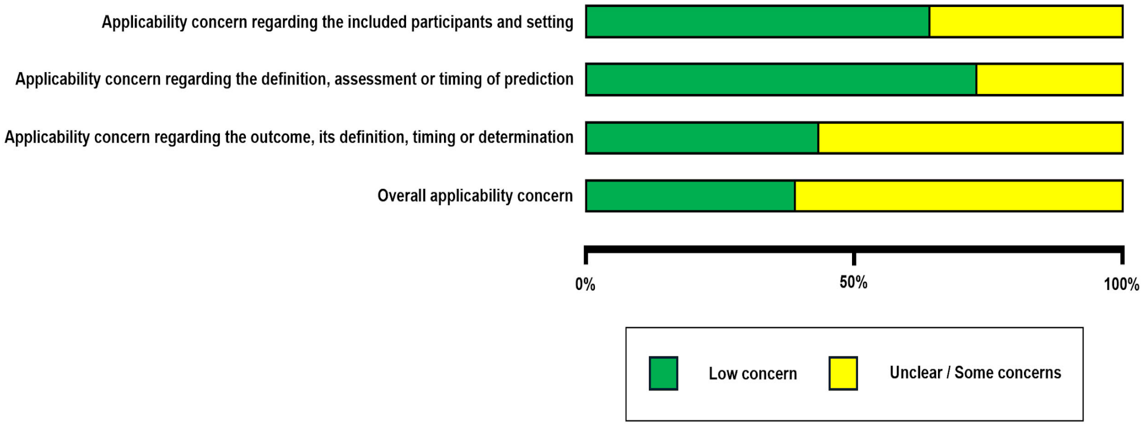

Supplementary Figure S2. An applicability analysis of the studies included is presented.

Supplementary Table S1 - Search queries across databases

Database	No	Search Query	Results
EMBASE			
	#1	#2 AND **'article'**/it<br>**#1** (**'artificial intelligence'**/exp OR **'artificial intelligence'** OR **'machine learning'**/exp OR **'machine learning'** OR **'deep learning'**/exp OR **'deep learning'** OR **'neural network'**/exp OR **'neural network'** OR **'artificial intelligence'**:ti,ab OR **'machine learning'**:ti,ab OR **'deep learning'**:ti,ab OR **'neural network'**:ti,ab OR **'radiomics'**:ti,ab OR **'predictive modeling'**:ti,ab OR **'computer assisted diagnosis'**:ti,ab) AND (**'spinal metastasis'**/exp OR **'spinal metastasis'** OR **'spine metastasis'**:ti,ab OR **'spinal metastasis'**:ti,ab OR **'metastatic spinal disease'**:ti,ab OR **'vertebral metastasis'**:ti,ab)	93
PubMed			
	#1	(("artificial intelligence"[MeSH Terms] OR "machine learning"[MeSH Terms] OR "deep learning"[MeSH Terms] OR "artificial intelligence"[Title/Abstract] OR "machine learning"[Title/Abstract] OR "deep learning"[Title/Abstract] OR "neural networks"[Title/Abstract] OR "radiomics"[Title/Abstract] OR "computer-assisted diagnosis"[Title/Abstract] OR "predictive modeling"[Title/Abstract]) AND ("spinal metastasis"[Title/Abstract] OR "metastatic spinal disease"[Title/Abstract] OR "spine metastasis"[Title/Abstract] OR "vertebral metastasis"[Title/Abstract] OR "metastatic spine disease"[Title/Abstract])) AND ((excludepreprints[Filter] OR medline[Filter]) AND (casereports[Filter] OR classicalarticle[Filter] OR clinicalstudy[Filter] OR clinicaltrial[Filter] OR multicenterstudy[Filter] OR observationalstudy[Filter] OR randomizedcontrolledtrial[Filter] OR technicalreport[Filter]) AND (humans[Filter]) AND (female[Filter] OR male[Filter]) AND (english[Filter]))	3
Scopus			
	#1	TITLE-ABS-KEY ("artificial intelligence" OR "machine learning" OR "deep learning" OR "neural network" OR "radiomics" OR "predictive modeling" OR "computer-assisted diagnosis" OR "AI-based diagnosis") AND TITLE-ABS-KEY ("spinal metastasis" OR "spinal metastases" OR "metastatic spinal disease" OR "spine metastasis" OR "vertebral metastasis") AND (LIMIT-TO (LANGUAGE , "English")) AND (LIMIT-TO (DOCTYPE , "ar"))	120
Web of Science			

	#1	TS=("artificial intelligence" OR "machine learning" OR "deep learning" OR "neural network" OR "radiomics" OR "predictive modeling" OR "computer-assisted diagnosis" OR "AI-based diagnosis") AND TS=("spinal metastasis" OR "spinal metastases" OR "metastatic spinal disease" OR "spine metastasis" OR "vertebral metastasis") and Article (Document Types)	83
Cochrane			
	#1	("artificial intelligence" OR "machine learning" OR "deep learning" OR "neural networks" OR "radiomics" OR "predictive modeling" OR "computer-assisted diagnosis" OR "AI-based diagnosis") AND ("spinal metastasis" OR "spinal metastases" OR "metastatic spinal disease" OR "spine metastasis" OR "vertebral metastasis")	5

Supplementary Table S1. Search queries across five databases (PubMed, Scopus, Web of Science Advance, Cochrane, and Embase (Ovid)) are shown.

Supplementary Table S2 – Summary of the studies analyzed

Sl No	Title	Year	Authors	Primary Tumor Type	Cohort Size	Type of Prediction Model	Output/ Prediction	Accuracy	Sensitivity	Specificity	AUC	Summary
**Quality Check**												
1	A prospectively deployed deep learning-enabled automated quality assurance tool for oncological palliative spine radiation therapy	2025	Kehayias, C.E. et al. [39]	primary tumor type is unknown	Retrospective cohort (n=513), Prospective cohort (n=400)	Skeletal Oncology Research Group Machine Learning Algorithm (SORG-MLA)	In all detected cases (i.e., 49 of 520 cases in total), the appropriate personnel were alerted. A false negative rate of 0·03% is estimated based on the 4% AI segmentation error rate and the frequency of reported spine radiation therapy errors.	NA	NA	NA	SORG-MLA: (AUROC 0.76, 95% CI 0.74–0.78) NESMS: (AUROC 0.66, 95% CI 0.64–0.68) METSSS: (AUROC 0.56, 95% CI 0.54–0.59).	By combining the rapid and accurate segmentation capabilities of the Total Segmentator model, performing consistency checks to reveal potential radiation therapy discrepancies, and following reporting protocols of individual health centers
**Complications – Blood Loss**												
2	Development and validation of a web-based artificial intelligence prediction model to assess massive intraoperative blood loss for metastati	2024	Shi, X et al. [15]	Thyroid cancer-4(2) prostate cancer-28(14.0), breast cancer-19(9.5), renal cancer-39(19.5), Lungcancer	200	LR and Machine learning	Logistic regression; K-nearest neighbor; Decision tree; XGBoosting machine; Random Forest; Support vector machine	Logistic regression (0.627); K-nearest neighbor (0.735); Decision tree (0.602); XGBoosting machine (0.771); Random Forest (0.723); Support vector machine (0.663)	NA	Logistic regression (0.643); K-nearest neighbor (0.643); Decision tree (0.452); XGBoosting machine (0.690); Random Forest (0.690); Support vector machine (0.667)	Logistic regression AUC (95% CI), 0.710 (0.665, 0.771); K-nearest neighbor 0.821 (0.786, 0.853); Decision tree 0.677 (0.636, 0.718);	The XGBM model may be a useful AI tool to assess the risk of intraoperative blood loss in patients with metastatic spinal disease undergoing decompressive surgery.

	c spinal disease using machine learning techniques			53(26.5), Hepatocellular carcinoma 12(6.0), Gastrointestinal system cancer 11(5.5), Urogenital cancer 7(3.5)							XGBoosting machine 0.857 (internal validation) (0.827, 0.877); Random Forest 0.826 (0.793, 0.861); Support vector machine 0.726 (0.693, 0.759)<br><br>XGBM external validation: 0.809 (0.778 – 0.860)	
5	Assessment of Hidden Blood Loss in Spinal Metastasis Surgery: A Comprehensive Approach with MRI-Based Radiomics Models	2024	Zhao, W. et al. [6]	Hypervascular-thyroid cancer, renal cell carcinoma, and hepatocellular tumors Nonhypervascular-none<br><br>Hypervascular-63(31.2%), Nonhypervascular-	202	Deep learning, MRI-based radiomics models	Hidden blood loss measure through T1WI radiomics model, FS-T2WI radiomics model, Fusion radiomics model	T1WI radiomics model 0.666 (0.644), FS-T2WI radiomics model 0.656 (0.654), Fusion radiomics model 0.765 (0.728).	T1WI radiomics model 0.693 (0.624), FS-T2WI radiomics model 0.712 (0.72), Fusion radiomics model 0.694 (0.678).	T1WI radiomics model0.656 (0.651), FS-T2WI radiomics model 0.636 (0.631), Fusion radiomics model 0.693 (0.672).	The fusion model AUC 0.744 (95% confidence interval [CI]: 0.576–0.914), T1WI model (AUC: 0.723, 95% CI: 0.542–0.906; P = 0.239) and the FS-T2WI model (AUC: 0.718, 95% CI: 0.544–	A radiomics model may serve as a promising assessment tool for the risk of HBL in patients undergoing spinal metastasis surgery, and guide perioperative planning to improve surgical outcomes.

				139(68.8%)							0.891; P = 0.181).	
16	Quantile regression-based prediction of intraoperative blood loss in patients with spinal metastases: model development and validation	2023	Li, J. et al. [40]	Nonvascular tumors included colon car-cinoma, non–small cell lung carcinoma, breast carcinoma, and sarcomas other than angiosarcoma and leiomyosarcoma, Hypervascular tumors included renal cell carcinoma, fol-licular thyroid carcinoma, neuroendocrine tumor, paragan-glioma, hepatocellular	528	Machine learning (quantile regression-based blood loss prediction model)	Hyper vascular tumor, higher BMI, and broader surgical extent were related with massive blood loss. Microwave ablation is more beneficial in surgery with substantial blood loss.	NA	NA	NA	NA	Machine learning models have greater predictive power and can offer useful tools to identify individuals with spinal metastatic disease who are experiencing severe psychological distress.

				carcinoma, leiomyosarcoma, angio-sarcoma, multiple myeloma, and melanoma. Nonvascular-254 (48.1) Moderate-145 (27.5) Hypervascular-129 (24.4)								
**Survival Prediction**												
3	Are Current Survival Prediction Tools Useful When Treating Subsequent Skeletal-related Events from Bone Metastases?	2024	Pan, Y.-T. et al. [28]	Spine or other extremity skeletal related events (humerus metastasis)	548	Skeletal Oncology Research Group machine learning algorithms (SORG-MLAs)	Survival rates, 42-day, 90-day, and 1-year survival rates were analyzed for the spine group, and 90-day and 1-year survival rates were analyzed for the extremity group	For spine group median Brier score was 0.11 (null Brier score 0.11) for 42-day survival, 0.18 (null Brier score 0.20) for 90-day survival, and 0.18 (null Brier score 0.20) for 1-year survival. For extremity group median Brier score	NA	NA	For spine group 42-day survival (median AUC 0.69 [95% CI 0.63 to 0.74], range 0.66 to 0.72), 90-day survival (median AUC 0.72 [95% CI 0.66 to 0.77], range 0.69 to 0.73),	The SORG-MLAs maintain satisfactory discriminatory capacity and offer considerable net benefits through decision curve analysis, indicating their continued viability as prediction tools in this clinical context.

								was 0.18 (null Brier score 0.18) for 90-day survival and 0.19 (null Brier score 0.22) for 1-year survival			1-year survival (median AUC 0.70 [95% CI 0.61 to 0.78], range 0.67 to 0.72). For extremity group 90-day survival (median AUC 0.65 [95% CI 0.57 to 0.72], range 0.64 to 0.67) and 1-year survival (median AUC 0.73 [95% CI 0.61 to 0.82], range 0.71 to 0.75).	However, the algorithms overestimate 1-year survival rates for patients with a subsequent SRE of the spine, warranting consideration of specific patient groups. Clinicians and surgeons should exercise caution when using the SORG-MLAs for survival prediction in these patients and remain aware of potential mispredictions when tailoring treatment plans, with a preference for less invasive treatments.
4	Survival in Patients with Spinal Metastatic Disease Treated Nonoper	2024	Fenn, B.P. et al. [26]	lung or liver metastasis-820 (39.5), brain metastasis-	415	Skeletal Oncology Research Group machine learning algorithms (SORG-MLAs)	90-day and 1-year mortality.	For 90-day mortality the Brier score was 0.15 (0.14, 0.16) relative to the null model Brier score	For 90-day mortality sensitivity was 0.82 (0.79, 0.85). For 1 year mortality sensitivity was 0.82	For 90-day mortality specificity was 0.73 (0.70, 0.75). For 1 year mortality specificity was 0.76	AUC of 0.85 (0.83, 0.87) and AUC=0.87 (0.85, 0.89) in the validation cohort	The SORG-ML algorithms for survival in spinal metastatic disease generalize well to patients

	atively with Radiotherapy: Are the SORG-ML Algorithms Relevant?			277 (13.4)				of 0.22. For 1 year mortality The Brier score was 0.15 (0.14, 0.16) relative to the null model Brier score of 0.24.	(0.80, 0.84).	(0.73, 0.79).	for 90-day and 1-year mortality, respectively	managed nonoperatively with radiation.
9	Development and internal validation of machine learning models for predicting survival in patients who underwent surgery for spinal metastases	2024	Santipas, B. et al. [12]	Breast cancer-79 (20.3), lung cancer-69 (17.7), prostate cancer, thyroid cancer, liver cancer-17 (4.4), hematologic malignancy	389	Machine learning	Survival outcomes at postoperative days 90, 180, and 365.	Linear discriminant analysis has highest accuracy 0.6691	NA	NA	k-fold cross validation 90 (CatBoost), 180 (XGBoost) and 365 days (XGBoost): 0.750, 0.726 and 0.731, testing 90 (CatBoost), 180 (XGBoost) and 365 days (XGBoost): 0.758, 0.744 and 0.693; 365 day - ROC AUC, 0.632–0.731), 180 day-ROC AUC, 0.654-0.726	These machine learning algorithms showed promising results in predicting survival in patients who underwent spinal palliative surgery for spinal metastasis, which may assist surgeons in choosing appropriate treatment and increasing awareness of mortality-related factors before surgery.
11	Does the Presence of Missing Data Affect	2024	Huang, CC et al. [27]	Slow growth - hormone-depend	2768	The Skeletal Oncology Research Group machine	Predict postoperative 6-week, 90-day, and 1-year survival probability of	NA	NA	NA	AUROC of 0.84 (95% CI 0.78 to 0.89) for 6-week	The SORG-MLA generally performed well in the

	the Performance of the SORG Machine Learning Algorithm for Patients with Spinal Metastasis? Development of an Internet Application Algorithm			ent breast cancer, hormone-dependent prostate cancer, malignant lymphoma, malignant myeloma, and thyroid cancer. Moderate growth tumor included non–small cell lung cancer with molecularly targeted therapy, hormone-independent breast cancer, hormone-independent prostate cancer, renal cell carcino		learning algorithm (SORG-MLA)	patients with spinal metastasis				prediction, 0.84 (95% CI 0.79 to 0.90) for 90-day prediction, and 0.77 (0.73 to 0.80) for 1-year survival prediction.	presence of one to three missing items, except for serum albumin level and lymphocyte count (which are essential for adequate predictions, even using our modified version of the SORG-MLA). We recommend that future studies should develop prediction models that allow for their use when there are missing data, or provide a means to impute those missing data, because some data are not available at the time a clinical decision must be made.

				ma, sarcoma, other gynecological cancer, and others. Rapid growth tumor included other lung cancer, colon and rectal cancer, gastric cancer, hepatocellular carcinoma, pancreatic cancer, head and neck cancer, other urological cancer, esophageal cancer, malignant melanoma, gallbladder cancer, cervical cancer,<br><br>Slow growth								

				-14 (86) Moderate growth - 31 (193) Rapid growth -55 (338) Brain metastases-19 (118) Visceral metastases-35 (218)								
15	A Machine Learning Algorithm for Predicting 6-Week Survival in Spinal Metastasis: An External Validation Study Using 2,768 Taiwanese Patients	2023	Su, C.-C. et al. [30]	Lung-1,130 (40.8) Breast-343 (12.3) Liver -279 (10.1) Prostate-213 (7.7) Colon/rectum -195 (7.0) Visceral metastases-993 (35.9) Brain metastases-567 (20.5)	2768	machine learning (SORG)	The 6-week survival rate was 84.2%, underestimation of the 6-week survival rate when the predicted survival rate is less than 50%.	good prediction accuracy with a Brier score of 0.11	NA	NA	prediction model achieved an AUC of 0.77 (95% CI, 0.74 to 0.79) in patients receiving only radiation therapy (n = 2,151), while achieving an AUC of 0.84 (95% CI, 0.79 to 0.90) in patients receiving surgery	The only tool can be used by both clinicians and patients in informative decision-making discussion before management of spinal metastasis.
18	External validation of the SORG	2023	Zhong, G. et al. [31]	Lung cancer, Visceral	150	SORG machine learning	The algorithm for 1-year mortality was	NA	NA	NA	AUC of the SORG ML	the SORG algorithms for predicting

	machine learning algorithms for predicting 90-day and 1-year survival of patients with lung cancer-derived spine metastases: a recent bi-center cohort from China			metastases-252 (34.4) Brain metastases-81 (11.1)		(ML) algorithm	well-calibrated with an intercept of 0.13 and a calibration slope of 1.00. However, the 90-day mortality prediction was underestimated with an intercept of 0.60 and a slope of 0.37.				algorithm for 90-day mortality prediction in lung cancer-derived spinal metastases is 0.714. (95%CI: 0.589-0.839) While the AUC for 1-year mortality prediction is 0.832 (95CI%, 0.758-0.906)	1-year mortality performed well on external validation. However, 90-day mortality was underestimated.
19	Evaluation of different scoring systems for spinal metastases based on a Chinese cohort	2023	Li, Z. et al. [29]	breast-52 (19.4%) Colon-5(1.9%) Esophagus-4 (1.5%) Stomach-5 (1.9%) Kidney-11 (4.1%) Liver-15 (5.6%) Lung-71 (26.5%) Leukaemia-1 (0.4%) Lymphoma-6 (2.2%)	268	Machine learning (SORG)		SORG ML scoring system demonstrated the highest accuracy	NA	NA	90-day survival after the index surgery in the present study, the SORG ML scoring system demonstrated the highest accuracy (AUC: 0.743) (0.666-0.817). The revised Katagiri (AUC: 0.711), VDL (AUC: 0.725), and SORG	None of the scoring systems can perform optimally at all time points and for all pathology types.

				Ovary-1(0.4%) Pancreas-2(0.7%) Prostate-13(4.9%) Rectum-10 (3.7) Sarcoma-7 (2.6%) Thyroid -12 (4.5%) Testicle-1(0.4%) Urinary tract-2(0.8%) Metastasis Liver or lung-99 (36.9%)- Brain-30 (11.2%)							nomogram (AUC: 0.722)<br><br>180-day) postoperative survival, the revised Katagiri demonstrated the highest accuracy (AUC: 0.761) (0.696-0.826), followed by the revised Tokuhashi (AUC: 0.723<br><br>1year: SORG ML AUC 0.787 (0.730-0.838)<br><br>2 years: Revised Katagiri AUC 0.779 (0.747-0.811)	
20	A Novel Prognostication System for Spinal Metastasis Patients Based on Network Science and	2023	Mezei, T. et al. [41]	Breast cancer, prostate cancer, thyroid cancer, angiosarcoma, chondrosarcoma, osteosa	454	Machine learning (COX model)	Five independent prognostic factors suitable for the design of the risk calculator was found. This new system has a better predictive ability	NA	NA	NA	0.706(95%CI 0.679to 0.733)	Accurate estimation of the life expectancy of cancer patients is essential for the implementation of personalised medicine. The training

	Correlation Analysis			r coma, Ewing sarcoma, bladder, colorectal, pharynx, kidney, liver, melanoma, ovarium, uterus, parotid, stomach,			compared with six other well-known systems with an average C-index of 0.706 at 10 years					performance of our system is encouraging, indicating the benefit of a network science-based visualization step.
22	Development and external validation of predictive algorithms for six-week mortality in spinal metastasis using 4,304 patients from five institutions	2022	Karhade, A.V. et al. [21]	Slow growth -881 (29.4) Moderate growth - 1035 (34.5) Rapid growth -1085 (36.2) Visceral Metastases- 1144 (38.1) Brain Metastases- 371 (12.4)	1303	Machine learning	The elastic-net penalized logistic model was chosen as the best performing model with AUC 0.84 on evaluation in the independent testing set	NA	NA	NA	Cross validation of training set Stochastic gradient boosting- 0.85(0.83 ,0.86) Random forest- 0.85(0.84 -0.86) Support vector machine- 0.77(0.75 -0.79) Neural network - 0.84(0.83 -0.86) Elastic net penalized logistic regression- 0.85(0.84	While this study does not advocate for the use of a 6-week life expectancy as criteria for considering operative management, the algorithm developed and externally validated in this study may be helpful for preoperative planning, multidisciplinary management, and shared decision-making in

											-0.86)<br><br>Testing: 0.84 (0.80-0.88)<br><br>External validation: 0.82 (0.78-0.85)	spinal metastasis
17	Can We Use Artificial Intelligence Cluster Analysis to Identify Patients with Metastatic Breast Cancer to the Spine at Highest Risk of Postoperative Adverse Events?	2023	Fourman, M.S. et al. [42]	Breast Cancer	202 patients	Machine learning (ECOG)	NON-COMP had the highest rate of lumbar metastatic tumors (68.4%) LOW/RADS had significantly lower SORG predictive 90-day (56.5 ± 22.2%) and 1-year (27.7 ± 17.7%) survival rates and the highest SORG 30-day mortality prediction (9.1 ± 7.7%)	NA	NA	NA	NA	Five distinct groups of patients that had different outcomes following open spine surgery for metastatic breast cancer. The highest performing patient clusters were those with a high functional level at the time of surgery (acute presentation) or with estrogen/progesterone positive metastatic disease.
23	The predictive ability of routinely collected laboratory markers for surgicall	2022	Li, Z. et al. [43]	lung (n = 71, 26.5%), breast (n=52, 19.4%), multiple myeloma	268	Skeletal Oncology Research Group machine learning (SORG ML)	optimal cutoff is 8.5×103/µL for WBC, 1.9×103/ µL for lymphocyte count, 3.9g/dL for albumin, and	Intra- and interobserver variability may also affect the prediction accuracy	NA	NA	90-day survival, the AUC of Tomita was 0.595 (95% CI, 0.494–0.687), the	Study confirmed that many routinely collected laboratory markers can serve as promising predictive

	y treated spinal metastases: a retrospective single institution study			(n=21, 7.8%),liver (n=15, 5.6%), Liver or lung metastasis-99 (36.9%) Brain metastasis - 30 (11.2%)			0.9mg/dL for creatinine.				revised Tokuhashi was 0.650 (95% CI, 0.548–0.745), the modified Bauer was 0.618 (95% CI, 0.518–0.708), the SORG ML was 0.743 (95% CI, 0.666–0.817)<br><br>1-year survival, the AUC of Tomita was 0.620 (95% CI, 0.552–0.689), the revised Tokuhashi was 0.702 (95% CI, 0.637–0.767), the modified Bauer was 0.646 (95% CI, 0.575–0.708), the SORG ML was 0.787 (95% CI,	factors for postoperative outcomes of patients with spinal metastasis

											0.730–0.838)	
26	Prognostic significance of lab data and performance comparison by validating survival prediction models for patients with spinal metastases after radiotherapy	2022	Yen, H.-K. et al. [32]	Lung cancer-1130 (40.8) Breast cancer-343 (12.3) Liver cancer-279 (10.1) Prostate cancer-213 (7.7) Colon/ Rectum cancer-195 (7.0) Visceral metastases-993 (35.9) Brain metastases-567 (20.5)	2786	Machine learning	Higher albumin, hemoglobin, or lymphocyte count was associated with better survival, while higher alkaline phosphatase, white blood cell count, neutrophil count, neutrophil-to-lymphocyte ratio, platelet-to-lymphocyte ratio, or international normalized ratio were associated with poor prognosis	NA	NA	NA	90-day-METSSS - 0.53(0.51 -0.55), NESMS-0.66(0.64 -0.68), SORG-0.78(0.76 -0.80) 1year-METSSS 0.56(0.54 -0.59), NESMS - 0.66(0.64 -0.68), SoRG-MLA-0.76(0.74 -0.78)	Laboratory data are of prognostic significance in survival prediction after RT for SM. Machine learning-based model SORG-MLA outperforms statistical regression-based model METSSS model and NESMS in survival predictions.
28	The performance of frailty in predictive modeling of short-term outcomes in the surgical management of metastatic tumors	2022	Bakhsheshian, J. et al. [7]	Lung cancer-,947 (12.2%) Breast cancer-908 (11.7%), Prostate cancer-1,199 (15.4%), Kidney cancer-	1974	Machine learning (Elixhauser Comorbidity Index)	Predictive modeling of frailty in subgroups demonstrated the greatest performance for mortality (AUC=0.750) in the lumbar spine, otherwise performed similarly for LOS, costs, complications, and discharge	NA	NA	NA	(mortality with an AUC of 0.644 and medical complications with an AUC of 0.671 MSTFI predictability performance for mortality	Frailty contributed to worse short-term adverse outcomes and could be more influential in the lumbar and junctional spine due to higher risk of deconditioning in the

	to the spine			687 (8.8%) Thyroid cancer-216 (2.8%), Sarcoma-88 (1.1%) Lymphoma/Myeloma-241 (3.1%) Melanoma-202 (2.6%) GI-334 (4.3%)			across subgroups. Outcomes analyzed included mortality, complications, length of stay (LOS), nonroutine discharges and costs.				decreased (AUC=0.565) when evaluating its external validity	postoperative period
29	Decreased psoas muscle area is a prognosticator for 90-day and 1-year survival in patients undergoing surgical treatment for spinal metastasis	2022	Hu, M.-H. et al. [44]	Lung (%)-70 (38.9%) Liver (%)-33 (18.3%) Breast (%)-19 (10.6%) Hematopoietic (%)-9 (5.0%) Kidney (%)-4 (2.2%) Others (%)-45 (25.0%)	180	Machine learning (SORG MLA)	PMA barely improved the discriminatory ability (c-index, 0.74; 95% confidence interval [CI], 0.67-0.82 vs. c-index, 0.74; 95% CI, 0.66-0.81)	NA	NA	NA	NA	Psoas muscle area is a prognosticator for 90-day survival and improves the discriminatory ability of earlier-proposed PSSs in our Asian cohort. However, incorporating PMA into more modern PSSs such as SORG-MLAs did not significantly improve its prediction performance.

30	A Prognostic Index for Predicting Survival of Patients Undergoing Radiation Therapy for Spine Metastasis Using Recursive Partitioning Analysis	2022	Walker, A. et al. [45]	visceral, brain, and bone metastases-228 (85%)	269	Machine learning		NA	NA	NA	NA	
35	Evaluating frailty, mortality, and complications associated with metastatic spine tumor surgery using machine learning-derived body composition analysis	2022	Massaad, E et al. [8]	Genitourinary 77 (25.4) Lung 60 (19.8) Gastrointestinal 43 (14.2) Breast 33 (10.9) Hematology 22 (7.3) Skin 15 (5.0) Bone sarcoma 13 (4.3) Head and neck 11 (3.6)	484	machine learning (deep learning pipeline)	The primary outcome measures were the 90-day and 1 year mortality risk, secondary outcome was the rate of major complications	NA	NA	NA	The model that included body composition and NESMS data showed significantly improved prediction of 1-year mortality compared with NESMS (AUC 0.73 and 95% CI 0.67–0.78 vs. AUC 0.70 and 95% CI 0.65–0.76,	body composition analyses that incorporate muscle mass, muscle density, and tissue adiposity are superior to those that include sarcopenia alone for predicting complications and mortality after surgery

				Thyroid 10 (3.3) Soft-tissue sarcoma 9 (3.0) Other 9 (3.0)								
36	International external validation of the SORG machine learning algorithms for predicting 90-day and one-year survival of patients with spine metastases using a Taiwanese cohort	2021	Yang, J.-J. et al. [34]	lung (n=153 [36%]), liver (n=54[13%]), breast (n=39[9.2%]), andprostate (n=25[5.9%]), VisceralMetastases-252(34.4), brain metastasis-81(11.1)	427	SORG Machine learning	1-year mortality had a calibration intercept of 0.08, 90-day mortality algorithm underestimated mortality for the lowest predicted probabilities, with an overall intercept of 0.81.	NA	NA	NA	External validation: 90d mortality: AUC 0.73 (0.67-0.78) ~~180d mortality~~ 1year mortality: AUC 0.74 (0.69-0.79)	The SORG algorithms for predicting 90-day and 1-year mortality in patients with spinal metastatic disease generally performed well on international external validation in a predominately Taiwanese population. However, 90-day mortality was underestimated
37	Updated external validation of the SORG machine learning algorithms for prediction of ninety-day and one-year mortality after	2021	Shah, A.A. et al. [33]	slow growth - Hormone-dependent breast Hormone-dependent prostate	298	machine learning (SORG)	At both postoperative time points, the SORG algorithms showed greater net benefit than the default strategies of changing management for no patients or for all patients.	NA	NA	NA	The AUC of the algorithms was 0.84 (95% confidence interval [CI]: 0.79–0.89) for 90-day mortality and 0.90 (95% CI:	With an independent, contemporary, and geographically distinct population, we report successful external validation of SORG algorithms for preoperativ

	surgery for spinal metastasis			Malignant lymphoma<br><br>Malignant myeloma<br><br>Thyroid,<br><br>Moderate growth Non-small cell lung<br><br>Hormone-independent breast Renal cell carcinoma<br><br>Sarcoma Other gynecological cancer Non-small cell lung rapid growth - Other lung Colon and/or rectal<br><br>Gastric<br><br>Hepato							0.86–0.93) for 1-year mortality	e risk prediction of 90-day and 1-year mortality after surgery for spinal metastasis.

				cellular carcinoma<br><br>Pancreatic Head and neck Other urological cancer<br><br>Malignant melanoma<br><br>Gallbladder<br><br>Cervical Slow growth 104(34.9)<br><br>Moderate growth 116(38.9) Rapid growth 78(26.2)<br><br>Visceral metastasis 104(35.0) Brain metastasis 23(7.8)								
40	A Novel Prediction Tool for	2020	He, X et al. [38]	subgroup 0: Lung, osteosa	265	Machine learning (COX)	This is a novel predicting model which	NA	NA	NA	AUC training and validatio	The input variables were found to be

	Overall Survival of Patients Living with Spinal Metastatic Disease			rcoma, stomach, bladder, esophagus, pancreas-94(50.8%),subgroup 1: Liver, gallbladder, unidentified-35(18.9%), subgroup 2: Others-10(5.4%), subgroup 3: Kidney, uterus-18(9.7%), subgroup 4: Rectum-5(2.7%), subgroup 5: Thyroid, breast, prostate, carcinoid tumor.-23(12.4%)			could generate a visualized predicted survival curve, and a table of the predicted survival rates at a series of time points, with no requirement of the complex calculation that performed in nomogram model. Moreover, the median survival time for an individual patient is also available in this widget.				n continuously > 0.750	significantly associated with the overall survival in patients living with spinal metastases. A user-friendly shiny app with favorable discrimination ability and consistency was released online for survival predicting.
41	Does the SORG algorith	2020	Bongers, M.E.R.	visceral metast	200	machine learning (SORG)	The SORG algorithms for 90-day	NA	NA	NA	External validation: 90d	The SORG algorithms for survival

	m generalize to a contemporary cohort of patients with spinal metastases on external validation?		et al. [35]	asis-127(63.5), brain metastsis-30(15)			and 1-year mortality retained good discriminative ability (c-statistic of 0.81 [95% confidence interval [CI], 0.74-0.87] and 0.84 [95% CI, 0.77-0.89])				mortality AUC 0.81 (0.74-0.87) ~~180d mortality~~ 1year mortality AUC 0.84 (0.77-0.89)	in spinal metastatic disease generalized well to a contemporary cohort of consecutively treated patients from an external institutional.
44	External validation of the SORG 90-day and 1-year machine learning algorithms for survival in spinal metastatic disease	2020	Karhade, A.V. et al. [36]	Lung, breast, prostate, renal. presence of visceral metastases (metastases in liver or lung)-49(27.8), presence of brain metastases-7(4.0), Three or more spine metastases-64(36.4)	176	machine learning (SORG)		NA	AUC of the SORG 90-day algorithm in the validation cohort was 0.81 and the AUC of the 1-year algorithm was 0.78	NA	Patients with complete data 90d-0.81(0.70-0.89), 1year-0.78(0.67-0.87)	Initial results from external validation of the SORG ML 90-day and 1-year algorithms for survival prediction in spinal metastatic disease suggest potential utility of these digital decision aids in clinical practice.
45	Development of Machine Learning Algorithms for	2019	Karhade, A.V. et al. [23]	Primary tumour type is unknown	1790	machine learning	The30-d mortality for the 1790 patients undergoing surgery for	NA	NA	NA	Training (k-fold cross validation): AUC 0.786	Machine learning algorithms are promising for

	Prediction of 30-Day Mortality after Surgery for Spinal Metastasis						spinal metastatic disease was 8.49%. machine learning algorithms developed to predict 30-d mortality performed well on discrimination (c-statistic), calibration (assessed by calibration slope and intercept), Brier score, and decision analysis.				Validation (split sample): 0.782	prediction of postoperative outcomes in spinal oncology and these algorithms can be integrated into clinically useful decision tools.
46	Predicting 90-Day and 1-Year Mortality in Spinal Metastatic Disease: Development and Internal Validation	2019	Karhade, AV et al. [24]	Other bone metastases 388(53)<br><br>Visceral metastases 252(34.4)<br>Brain metastases 81(11.1)<br>Tumor location<br><br>Cervical 104(14.2)<br><br>Thoracic	732	machine learning (SGB)	the primary outcomes consisted of 90-d and 1 yr mortality. Ninety-day and 1-yr mortality could be ascertained in 722 (98.6%) and 709 (96.9%) patients, respectively.	NA	NA	NA	Stochastic gradient boosting: Training (k-fold cross validation): 0.83 (0.81-0.85)-90d 0.85 (0.83-0.87)-1yr<br><br>Testing; 0.83 90d, 0.89 1y	Preoperative estimation of 90-d and 1-yr mortality was achieved with assessment of more flexible modeling techniques such as machine learning.

				425(58.1)<br><br>Lumbar 164(22.4)<br><br>Multiple 39(5.3)								
47	Development of a prognostic survival algorithm for patients with metastatic spine disease	2016	Pereira, N.R.P. et al. [25]	Lung, kidney, breast, hematological, prostate, melanoma, colorectal, neuroendocrine, sarcoma, head and neck, thyroid, liver, esophageal, endometrial, bladder, pancreatic, gynec, gastric, other, unknown, Other bone	649	machine learning (boosting algorithm)	We compared the performance of the nomogram between patients with good prognosis tumors (n = 431) and bad prognosis tumors (n = 218), and found no substantial differences.	0.76 (30 days), 0.74 (90 days), and 0.77 for 365-days with relatively reliable out-of-sample (test dataset) estimations.	98.50%	98.90%	Training (k-fold cross validation) Boosting Algorithm: AUC 30d 0.91 (0.86-0.95), 90d 0.86 (0.83-0.90), 1y 0.84 (0.80-0.87)<br><br>Testing (Nomogram): AUC 30d 0.75 (0.60-0.89), 90d 0.73 (0.63-0.83), 1y 0.75 (0.67-0.84)<br><br>0.70(0.64 to 0.76) for 30-	A nomogram proved to be an accurate tool to predict survival and can be made available on web-based applications to assist the surgeon in decision-making.

				metastases outside of the spine 342(53)<br><br>Visceral metastases at time of surgery None 405(62) Liver or lung 172(27) Brain 29(4)<br><br>Liveror lungandbrain 43(7)							days, 0.69 (0.65 to 0.73) or 90-days, and 0.73 (0.69 to 0.77) for 365-days for both the training (in-sample) as the test datasets (out-of-sample)	
**Complications: Non-specific**												
6	Machine learning–based detection of sarcopenic obesity and association with adverse outcomes in patients undergoing surgical treatment for spinal metastases	2024	Khalid, S.I. et al. [46]	Hematological Breast cancer-7 (11), GI cancer-5 (8.1) , GU cancer-18 (29) , head and neck cancer-4 (6.5), lung cancer-10 (16) ,	62	Automated machine learning pipeline	Measures of frailty (MISTFI p = 0.36; mFI-5 p = 0.86), and ambulatory status before surgery (p = 0.54) were not significantly different between the two groups.	NA	NA	NA	Hazard ratio mentioned	The SO phenotype was associated with increased odds of non-home discharge, readmission, and postoperative mortality. This study suggests that SO may be an important prognostic factor to consider when developing

				thyroid cancer-2 (3.2) , bone sarcoma-4 (6.5) , sarcoma-2 (3.2) , skin cancer-5 (8.1)								care plans for patients with spinal metastases.
8	Developmental and Validation of Machine Learning Model for Prediction Complication after Cervical Spine Metastases Surgery	2025	Santipas, B. et al. [11]	Primary tumor site Lung 21(29.17) Breast 20(27.78) Other 31(43.05) Visceral metastases 25.42 Lung 18(25) Liver 16(22.22)	72	Machine learning. Predictive models were developed using Gradient Boosting, Logistic Regression, and Decision Tree Classifier algorithms.	AUC, F1-score, precision, recall, and SHAP values	NA	NA	NA	The Gradient Boosting model had the best performance with an AUC of 0.939 (training, k-fold cross validation) and 0.873 (validation), decision training - 0.917 and 0.718, logistic regression -0.715 and 0.645 (under TRAINING SET and TEST SET respectively)	The Gradient Boosting machine learning model showed superior performance in predicting postoperative complications in cervical spine metastases surgery. With continuous data updating and model training, machine learning can become a vital tool in clinical decision-making, potentially improving patient outcomes.
13	Implication of nutritional status	2023	Rigney, G.H. et al. [47]	Bone sarcoma-9 (5.8%)	154	Machine learning	Postoperative survival and complication rates, with	NA	NA	NA	% survival after 3 months	The PNI was most predictive of

	for adverse outcomes after surgery for metastatic spine tumors			, breast cancer-15 (9.7%) colorectal cancer-7 (4.5%) haematological cancer-17 (11.0%) , renal cancer-20 (13.0%) , lung cancer-23 (14.9%), prostate cancer-20 (13.0%), other			focus on wound-related complications				Group IV-93.5(89.3,100) GROUP III-86.1(72.7,100) GROUP II-73.5(60.1,90) GROUP I-12.5 (3.4,45.7) %survival after 6 months GROUP IV-88.4(79.3,98.5) GROUP III-71.8(54.9,93.8) GROUP II-58.8(44.4,77.9) GROUP I-6.3(0.9,41.7)	complications and may be a key biomarker for risk stratification in the 90 days following surgery. Nutrition consultation was associated with a reduced risk of wound-related complications, attesting to the importance of this preoperative intervention. These findings suggest that nutrition plays an important role in the postsurgical course and should be considered when developing a treatment plan for spinal metastases.
39	Performance assessment of the metastatic spinal tumor frailty	2021	Massaad, E. et al. [10]	Lung 90 (18.8) Kidney 51 (10.7) Breast 47 (9.8)	479	machine learning (random forest)	The occurrence of at least 1 major postoperative complication within 30 of surgery	NA	0.21 95% CI 0.14–0.28 in the reference model vs. 0.37, 95% CI 0.29–0.45	0.90, 95% CI 0.87–0.93) in reference model compared with the random forest	in-hospital mortality (AUROC 0.69, 95% CI 0.54–0.85) in the	The importance of externally validating prediction models developed from

	index using machine learning algorithms: limitations and future directions			Blood (heme) 44 (9.2) Prostate 42 (8.8) Colon/rectal 28 (5.9) Bone sarcoma 24 (5.0) Thyroid 23 (4.8) Head & neck 22 (4.6) Skin 21 (4.4) Other 86 (17.9)					in random forest	machine learning model (0.87, 95% CI 0.83–0.90	validation cohort. AUROC 0.62, 95% CI 0.56–0.68 for random forest (training), AUROC 0.69 (0.66-0.73) (internal bootstrap validation) vs. AUROC 0.56, 95% CI 0.50–0.62 for logistic regression poor dis-crimination for predicting complications (AUROC 0.56, 95% CI 0.50–0.62	national databases, and recognizing inherent methodological limitations with prognostic model development as significant associations between MSTFI classes and outcomes wasn't observed
**Complications – Venous Thromboembolism**												
7	Development of Machine Learning Algorithms for Predicting Preoperative and Postoperative Venous	2024	Santipas, B. et al. [13]	Lung cancer 92 (27.5), breast cancer 58 (17.4), prostate cancer- 43 (12.9),	334	Machine learning	VTE prevalence within 30 and 90 days, with pulmonary embolism (PE) and deep vein thrombosis (DVT) rates at 20% and 80%, respectively.	The rando forest algorithm was the best-performing MLM for predicting VTE within 90 days, with accuracy values of	NA	NA	30 days prediction Pre op- 0.62 Post op- 0.62 90 days prediction Pre op- 0.69	Predictive analytics and MLMs effectively predict preoperative and postoperative VTE in patients undergoing surgery for spinal

	Thromboembolism in Patients Undergoing Surgery for Spinal Metastasis			liver cancer-21 (6.3), renal cancer-17 (5.1), colorectal cancer-14 (4.2), thyroid cancer-14 (4.2), haematological cancer-12 (3.6), cholangiocarcinoma-11 (3.3), nasopharyngeal cancer-9 (2.7),cervical cancer-6 (1.8), other				0.88 preoperatively and 0.86 postoperatively. For predicting VTE within 30 days, the support vector machine model was most effective, with accuracy of 0.88 preoperatively and 0.59 postoperatively			Post op-0.67<br><br>Gradient Boosted Trees (Testing – k-fold cross validation): preop 30d 0.77, postop 30d 0.71,<br><br>Support Vector Machine (Testing – k-fold cross validation): preop 90d 0.72, Postop 90d 0.68,	metastasis. Identified key factors and MLM performance metrics offer valuable insights for risk assessment and preventive measures in this patient population
**Post-operative Ambulatory Status**												
10	Establishment and validation of an interactive artificial intelligence platform to predict postoperative	2024	Cui, YP et al. [12]	Thyroid cancer-5(2.3), prostate cancer 31(14.1) breast cancer-20(9.1), renal cancer 43(19.	445	Machine learning (extreme gradient boosting machine (eXGBM), sup- port vector machine (SVM), random forest (RF), neural network	The primary outcome of this study was the ambulatory status of the patients within one week after surgery.	Neural network has the highest accuracy of 0.861		eXtreme gradient boosting machine has the highest specificity of 0.843	Ensemble Model: k-fold cross validation: 0.911 (0.854-0.968) External validation 1: AUC 0.873 (0.809-0.936) External	The study successfully develops and validates an interactive AI platform for evaluating the risk of postoperative loss of ambulatory ability in patients

	ambulatory status for patients with metastatic spinal disease: a multi-center analysis			5), lung cancer 58(26.4), hepatocellular cancer 13(5.9), gastrointestinal cancer 12(5.5), urogenital cancer-8(3.6), opthers-30(13.6)		(NN), and decision tree (DT))					validation 2: AUC 0.924 (0.890-0.959)<br><br>AUC Random forest model has highest AUC of 0.926 (0.903–0.942)	with metastatic spinal disease. This AI platform has the potential to serve as a valuable model for guiding healthcare professionals in implementing surgical plans and ultimately enhancing patient outcomes.
21	Development of Machine Learning Models to Predict Ambulation Outcomes Following Spinal Metastasis Surgery	2023	Chavalparit, P. et al. [16]	Breast cancer -41 (29.9), thyroid cancer -10 (7.3), kidney cancer -5 (2.9), liver cancer - 4 (2.9), prostate cancer -12 (8.8), lung cancer-21 (15.3), hematological, cholangio,	167 405	deep learning	90-day ambulatory outcome-(AUC, 0.94; accuracy, 0.82; precision, 1; recall, 0.79; and F1-score, 0.88), 180-day ambulatory outcome-(AUC, 0.85; accuracy, 0.82; precision, 0.82; recall, 1; F1-score, 0.9)	180d- 0.82, 90d- 0.82	NA	NA	Testing (k-fold cross validation) 180d extreme gradient boosting: AUC 0.852 (Extreme Gradient Boosting)<br><br>Testing 90d (k-fold cross validation) decision tree: AUC 0.941 (Decision Tree)<br><br>Developed 13 ML	The extreme gradient boosting and decision tree algorithms best predicted postoperative ambulatory status 180 and 90 days after spinal metastasis surgery, respectively.

				nasopharyngeal, colorectal, cervical, unkown, others							algorithms and identified the best predictive model for ambulatory status 180 (extreme gradient boosting) and 90 (decision tree) days following surgery with AUC values of 0.85 and 0.94, respectively.	
**Treatment Outcome**												
12	MRI feature-based radiomics models to predict treatment outcome after stereotactic body radiotherapy for spinal metastases	2023	Chen, Y. et al. [17]	lung cancer-49(34.5%), abdominal cxanmcer-48(33.8%), others-45(31.7%)	194	Machine learning	Prediction models for patients with spinal metastases undergoing stereotactic body radiotherapy (SBRT).	Highest accuracy is ALL+ Clinical 0.830	highest sensitivity is for FS-T2WI + Clinical 0.598	highest specificity is FS-T2WI 0.972	k-fold cross validation ALL (T1WI + T2WI + FS-T2WI): AUC 0.828<br><br>Optimal models constructed based on T1WI, T2WI, and FS-T2WI sequences achieved AUC values of 0.779 (QDA), 0.823	MRI is commonly utilized as an imaging modality before SBRT, enabling a comprehensive evalu-ation of target lesions. Through the analysis of radiomic features in MRI, our constructed models could predict treatment outcomes following SBRT in spinal

											(GP), and 0.745 (QDA) respectively. radiomics models con-structed using MRI demonstrate better performance (AUC = 0.745–0.825)	metastases. Furthermore, the incorporation of clinical features further improved the performance of the models. .
24	Radiomics analysis based on multiple parameters MR imaging in the spine: Predicting treatment response of osteolytic bone metastases to chemotherapy in breast cancer patients	2022	Shi, Y.-J. et al. [9]	breast ~~and bone cancer~~	36	Machine learning	The radiomics based on ADCall had the highest AUC value (0.852), followed by that of the T2WI (0.829) and FS-T2WI (0.798). The radiomics model using ADCall and FS-T2WI showed excellent efficiency in predicting treatment response	NA	NA	NA	ADCall + T2WI Training: AUC 0.908 (0.86-0.96) ADCall + FST2WI Validation: AUC 0.873 (0.78-0.96)<br><br>Training cohort T1WI-0.776 (0.69–0.87) T2WI-0.829 (0.76–0.90) FST2WI-0.798 (0.72–0.88) ADCall-0.852 (0.79–0.92)	Radiomics model based on ADCall and FS-T2WI could predict the treatment response and contribute to assisting clinicians in accurately choosing appropriate management.

											ADC0–800- 0.740 (0.65–0.83) DDC- 0.783 (0.70–0.86) D- 0.811 (0.74–0.87)<br><br>Validation cohort T1WI- 0.607 (0.46–0.75) T2WI- 0.686 (0.54–0.83) FST2WI- 0.795 (0.68–0.91) ADCall- 0.809 (0.70–0.92) ADC0–800- 0.749 (0.62–0.88) DDC- 0.678 (0.54–0.82) D- 0.770 (0.64–0.90)<br><br>.	
25	Proposing a quantitative MRI-based linear measurement	2022	Jabehdar Maralani, P. et al. [20]	Tumor location<br><br>Cervical -46 (7.8)<br><br>Thorac	323	Machine learning (Decision tree)	Compared to baseline, an increase of > 3 mm in any lesion dimension, combined with a 1.67-fold increase	93.2%	96.8%	89.6%	Training (k-fold cross validation) Model- 0.923, Test	593 spinal segments treated with SBRT investigated. Through recursive portioning analysis a

	framework for response assessment following stereotactic body radiation therapy in patients with spinal metastasis			ic -340 (57.3)<br><br>Lumbar -166 (28)<br>Sacral - 41 (6.9)<br>Tumor Type<br>Lytic - 262 (44.2)<br><br>Sclerotic- 192 (32.4)<br>Mixed -48 (8.1)<br><br>Unknown - 91 (15.3)			in area, had an OR of 4.6 for SSP.				model- 0.959	clinically feasible model for response assessment guidelines was developed.
27	Deep Learning Model for Classifying Metastatic Epidural Spinal Cord Compression on MRI	2022	Hallinan, J.T.P.D. et al. [48]	breast-23(17.8), lung cancer-21(16.3), prostate-19(14.7), colon-15(11.6), renal carcinoma-10(7.8), Nasopharyngeal carcinoma-9(7), others-32(24.8)	164 patients (215 MRI studies, 177 training / validation, 38 internal testing) 174	Deep learning (MESCC)	DL model showed almost-perfect agreement (k = 0.92, p < 0.001) for dichotomous Bilsky classification (low grade versus high grade), similar to specialist readers (k = 0.96–0.98, all p < 0.001)	NA	DL model Internal dataset- 97.6(91.7-99.7), external dataset- 89.9(84.4-94)	DL model Internal dataset- 93.6(91.9-95), external dataset- 98.1(96.7-99.1)	NA<br><br>DL model overall- 0.94	DL model is reliable and may be used to automatically assess the Bilsky classification of metastatic epidural spinal cord compression on thoracic spine MRI. The DL model used to triage MRI scans for urgent reporting, augment non-sub-specialized radiologists, improve the

												communication and referral pathways between oncology, surgery specialists.
31	Deep Learning Model for Grading Metastatic Epidural Spinal Cord Compression on Staging CT	2022	Hallinan, J.T.P.D. et al. [19]	lung cancer-36(23.2), breast cancer-33(21.3), colon cancer-15(9.7), prostate cancer-13(8.4), renal carcinoma-12(7.7), multiple myeloma-10(6.5), Hepatocellular carcinoma - 8(5.2), Nasopharyngeal carcinoma-6(3.9), others	185	Deep learning (MESCC Classification)	breast and lung malignancies the most frequent cancer subtypes (86/185 patients, 46.5%). The majority of thoracic MESCC was located at the thoracolumbar border between T11-L3 (75/185 patients, 40.5%)	NA	88%	89%	Testing: DL model showed high AUCs ranging from 0.953 (95% CI 0.934–0.971) for the SWL spine window to 0.971 (95% CI 0.961–0.981) for the SWL max fusion model (Bilsky grading (normal/low versus high)) Testing: SWL spine-window AUC 0.924 (0.910-0.938) (Bilsky grading (normal versus low/high))	a deep learning (DL) model for the Bilsky grading of metastatic epidural spinal cord compression on staging CT studies. The deep learning model had a superior interobserver agreement for the detection of trichotomous Bilsky grading (normal, low, and high-grade) compared to general and specialist radiologists.

											superior performance compared to all readers including FEM and HYO with the best human performance (AUC = 0.891, 95% CI 0.863–0.918 and AUC = 0.891, 95% CI 0.863–0.919, respectively)	
33	Clinical utility of convolutional neural networks for treatment planning in radiotherapy for spinal metastases	2022	Arends, S.R.S. et al. [49]	Primary tumor type is unknown	59	Deep learning	Better segmentation performance was seen for lumbar vertebrae (DSC difference: between +1.4% and +2.6%, HD difference: between +0.1 and –1.1, compared to thoracic vertebrae).	The sequential approach achieved 91% and 80% accuracy on the internal and external validation, respectively.	NA	NA	NA	Found a feasible approach for automatic vertebral body delineation using two variants of a multi-scale CNN. This approach generates high quality automatic delineations
34	Evaluation of a Dedicated Software “Elemen	2022	Rogé, M. et al. [50]	Breast cancer ,10 (33%) lung cancer-	30	deep learning	[HIBrainLab = 0.18 ± 0.06 versus HIExpert of 0.17 ± 0.06 (p = 0. 916)],	For metastases that involved the vertebral	NA	NA	NA	A solution for semiautomatic segmentation of CTVs

	ts™ Spine SRS, Brainlab®" for Target Volume Definition in the Treatment of Spinal Bone Metastases with Stereotactic Body Radiotherapy			,8 (27%) prostate cancer 8 (27%), others - 4(13)%, adenocarcinoma- 17(56%), ducal carcinoma- 8(27%)			[PCIBrainLab = 1.33 ± 0.15 versus PCIExpert = 1.24 ± 0.12 (p = 0.217)], [GIBrainLab = 4.47 ± 0.41 versus GIExpert 4.07 ± 0.49 (p = 0.062)]	body only (n = 13, 37%), the mean DSC was 0.90 ± 0.03. The DSC for metastases that involved the vertebral body only was on average 0.09				for spine metastases treated with SBRT. The results showed that the semiautomatic method had quite good accuracy and can be used in clinical routine even for complex lesions.
38	Stereotactic body radiation therapy for spinal metastases: A novel local control stratification by spinal region	2021	Kowalchuk, R.O. et al. [51]	Breast cancer 69 (36), prostate cancer - 21(11), non-small cell lung cancer - 29(15), others- 71(37)	165	Machine learning	72% of patients had improvement of pain after SBRT, while 14% were stable and only 5% felt increased pain	NA	NA	NA	NA	Patients with sacral spine lesions may have decreased local control due to increased PTV
42	A Validated T Cell Radiomics Score Is Associated with Clinical Outcomes Following Multisite SBRT	2020	Korpics, M.C. et al. [52]	Ovarian/fallopian tube cancer -8 (15.7), non-small cell lung cancer - 6 (11.8), Breast	68	deep learning	Higher RS was associated with improved PFS (HR 0.12; 95% CI, 0.03- 0.51; P Z .004), but its association with TMC (HR 0.36, 95% CI, 0.02-7.02; P Z .502) and	NA	NA	NA	NA	clinical validity of the RS (at the 25% percentile cutoff) as a prognostic biomarker in patients treated with SBRT þ P. Further clinical validation studies of

	and Pembrolizumab			cancer - 2 (3.9), Cholangiocarcinoma - 4 (7.8), Endometrial cancer -6 (11.8) Colorectal cancer - ,3 (5.9) Head and neck cancer-4 (7.8)			OS (HR 0.28, 95% CI, 0.05-1.55; P Z .144)					the RS in the setting of combined radiation therapy and immunotherapy
43	The first algorithm calculating cement injection volumes in patients with spine metastases treated with percutaneous vertebroplasty	2020	Cui, Y. et al. [53]	Primary tumor type is unknown	251	Machine learning	Group C (28.6%) had the highest rate of cement leakages than patients in group A (14.4%) and B (23.5%) (P=0.014). In addition, vertebrae in group C (14.3%) were more prone to intra-spinal canal leakage, as compared with vertebrae in group A (1.2%) and B (2.3%).	NA	NA	NA	Testing: The AUROC value for the variable of vertebrae collapse alone was 0.60, the variable of groups alone was 0.69, and Bilsky scale alone was 0.71. When the three variables combined, the AUROC value could increase to 0.73.	The algorithm can facilitate surgical planning and guide cement injection. Bilsky scale is an independent risk factor for intra-spinal canal cement leakages.

**Complications – Psychological**												
14	Machine learning-based algorithms to predict severe psychological distress among cancer patients with spinal metastatic disease	2023	Gao, L. et al. [18]	Lung cancer -605 (58.01 %), liver cancer 49 (4.70%), gastrointestinal cancer- ,78 (7.48%) breast cancer -87 (8.34%), others-224(21 .48%)	1043	machine learning (XGBoosting machine, random forest, gradient boosting machine, support vector machine, and ensemble prediction model, as well as a logistic regression model)	Up to 21.48% of all patients who were recruited had severe psychological distress<br><br>the AUC of the logistic regression model was only 0.836 (95% CI: 0.756-0.916; Accuracy: 0.783).	NA	NA	NA	Testing: Gradient boosting machine algorithm: AUC of 0.865 (95% CI: 0.788-0.941) XGBooting machine algorithm (AUC: 0.851, 95% CI: 0.768-0.934; Ensemble prediction (AUC: 0.851, 95% CI: 0.770-0.932)	Machine learning models have greater predictive power and can offer useful tools to identify individuals with spinal metastatic disease who are experiencing severe psychological distress.
**Complications – Vertebral Body Related**												
32	Radiomic modeling to predict risk of vertebral compression fracture after stereotactic body radiation therapy for spinal metastases	2022	Gui, C. et al. [22]	prostate cancer - 17(17.9), renal cell cancer-13(13.7), non small cell lung cancer-12(12.6), colorectal-10(10.5), breast - 8(8.4), sarcoma-	74	Machine learning	The most accurate classification model which included both radiomic and clinical features significantly outperformed models that included clinical features alone or only components of the SINS	NA	0.956	0.262	Best performing model-0.878 (0.832–0.924), SINS-0.579 ((0.496–0.663) Radiomic features -0.872 (0.828–0.915)	Findings remain to be validated using a Multi institutional dataset but may ultimately aid physicians in objectively assessing a given patient’s risk of radiation-induced VCF based on routinely available

				6(6.3), other								clinical data

Supplementary Table S2. A summary of the studies focusing on genetic and molecular signatures in spinal metastases is presented.
